# How family functioning shapes prenatal bonding: a mediation analysis of depressive symptoms and maternal–fetal attachment

**DOI:** 10.3389/fpsyt.2026.1800384

**Published:** 2026-04-22

**Authors:** Silvia Mammarella, Laura Giusti, Laura Camoni, Fiorino Mirabella, Manuela Ludovisi, Rita Roncone

**Affiliations:** 1 Department of Life, Health and Environmental Sciences, University of L’Aquila, L’Aquila, Italy; 2 Center for Behavioural Sciences and Mental Health, National Institute of Health, Rome, Italy; 3 Unit of Obstetrics and Gynecology, San Salvatore Hospital, L’Aquila, Italy; 4 Rehabilitation Treatment, Early Interventions in Mental Health University Unit —San Salvatore Hospital, L’Aquila, Italy

**Keywords:** depressive symptoms, family functioning, maternal–fetal attachment, mediation model, perinatal mental health, pregnancy

## Abstract

**Objective:**

Pregnancy is a period in a woman’s life characterized by profound hormonal, psychological, and social changes, which may lead to increased emotional vulnerability, during which depressive symptoms may negatively affect maternal well-being and maternal–fetal attachment. Although family functioning has traditionally been examined mainly in relation to psychopathology, its protective role during pregnancy remains insufficiently explored. This study examined whether depressive symptoms mediate the association between family functioning and antenatal maternal attachment.

**Methods:**

The sample consisted of 261 pregnant women enrolled in a multicenter Italian perinatal mental health project. The present add-on study focused specifically on family functioning, social support, and maternal antenatal attachment. Participants completed self-report measures assessing depressive symptoms (Edinburgh Postnatal Depression Scale), maternal–fetal attachment (Maternal Antenatal Attachment Scale), family functioning (Family Functioning Questionnaire), perceived partner support, and social network quality. Structural equation modeling was used to test a mediation model in which depressive symptoms mediated the relationship between family functioning and two attachment dimensions: quality of attachment and intensity of preoccupation. Models were estimated using full-information maximum likelihood with bootstrapped confidence intervals.

**Results:**

Higher family functioning was significantly associated with lower depressive symptom severity. Depressive symptoms were negatively associated with attachment quality but not with intensity of preoccupation. A small but significant indirect effect of family functioning on attachment quality, mediated by depressive symptoms, emerged, indicating partial mediation. No mediation effect was observed for intensity of preoccupation, which showed a direct positive association with family functioning. These results remained substantially unchanged after controlling for perceived partner support and social network quality.

**Conclusion:**

Depressive symptoms partially mediate the relationship between family functioning and the emotional quality of maternal–fetal attachment, whereas the intensity of preoccupation appears to be more directly linked to the broader family environment. These findings underscore the importance of assessing family functioning during pregnancy and suggest that interventions targeting both family dynamics and maternal depressive symptoms may foster healthier prenatal attachment.

## Introduction

Pregnancy is a period in a woman’s life characterized by profound hormonal, psychological, and social changes (1–4), which may increase emotional vulnerability (5). The biopsychosocial approach provides an essential theoretical framework by integrating the biological, psychological, and social dimensions of the perinatal period, thereby shaping both maternal and fetal health. Consistent with this perspective, social neuroscience has shown that attachment and emotion regulation processes are rooted in specific neurobiological circuits that are highly sensitive to interpersonal contexts (6, 7).

Among antenatal psychological complications, depressive symptoms play a major role, with an estimated global prevalence of approximately 20% (8). In a recent study of pregnant women from Southern Italy, 11.9% exhibited symptoms of perinatal depression. Women at risk also reported greater impairment in psychological functioning and quality of life, along with relational difficulties with both their family of origin and their partner. More specifically, women at higher risk for perinatal depression displayed elevated levels of relational anxiety and avoidance. These insecure attachment dimensions were associated with increased psychological vulnerability during the perinatal period, highlighting attachment as a significant relational factor in the risk for perinatal depression (9).

According to Bowlby’s attachment theory, early significant relationships with primary caregivers (typically parents) contribute to the development of internal working models that shape how individuals perceive themselves and others in terms of security, reliability, and availability of emotional support (10). In clinical contexts, women with insecure attachment often experience greater difficulties in intimate and family relationships, as their internal models derived from the birth family influence emotional regulation, perceived social support, and vulnerability to mood disorders, including perinatal depression (11, 12). In line with these findings, women with perinatal depression report significantly higher levels of conflict with both their birth family (29.2% vs. 7.9%, p < 0.001) and their partners (32.6% vs. 4.5%, p < 0.001) compared to healthy controls (11). The functioning of the birth family plays a critical role in shaping these internal models: a family characterized by support, cohesion, and positive communication promotes the development of a secure attachment style, whereas recurrent conflict, rejection, ambivalence, or emotional distance fosters insecure attachment styles (avoidant, anxious-ambivalent, or disorganized) (10).

Depressive symptoms during pregnancy negatively affect not only maternal mental health and well-being, but also fetal and neonatal development, as well as maternal–fetal attachment (13–16). Importantly, longitudinal studies have shown that stronger attachment during pregnancy predicts more optimal infant and toddler outcomes (17). Although the existing literature remains limited and findings are sometimes inconsistent, this area warrants further investigation (18).

Regarding factors influencing maternal antepartum mood, good family functioning—defined as satisfaction with family communication and problem-solving skills, and support for individual goals within the family—is a key protective factor. Family functioning has long been a central and controversial topic in discussions of its influence on the mental health of one of its members, particularly in severe mental disorders, with families frequently portrayed in negative terms. More recently, research in mental health has increasingly focused on families’ positive resources and strengths (19).

In pregnant women, positive family functioning was found to be negatively associated with depressive symptom severity (20). Empirical evidence indicates that family functioning and perceived social support exert both direct and indirect effects on prenatal depression and maternal self-efficacy (12, 21). Moreover, growing evidence highlights a significant association between depressive symptoms, partner/family/social support, and maternal-fetal attachment (22–24).

However, relatively few studies have examined how overall family functioning simultaneously contributes to maternal depressive symptoms and antenatal attachment, or whether depressive symptoms may act as a psychological mechanism linking family functioning to attachment outcomes. Examining these dynamics within an integrated framework may help clarify the mediating role of depressive symptoms and inform the development of targeted preventive interventions aimed at promoting perinatal mental health and strengthening early relational bonds.

The present study tested a mediation model in which depressive symptoms were hypothesized to mediate the association between family functioning and two dimensions of antenatal maternal attachment: attachment quality and preoccupation with fetal health.

## Materials and methods

### Design

The present study is part of an ongoing, multicenter project coordinated by the Italian National Institute of Health (ISS). Launched in November 2021, the project aims to monitor symptoms of anxiety and depression from the outset of pregnancy until 12 months after delivery. The Italian Network for Perinatal Mental Health currently includes thirteen public health facilities, including obstetrics and gynecology departments, maternal and child health services, and hospital psychiatry departments, distributed across eight Italian regions. All centers use the same screening tool (Edinburgh Postnatal Depression Scale, EPDS) (25) and data collection and referral protocols developed by the ISS.

The present add-on study focused specifically on maternal antenatal attachment, family functioning, and social support, and included the following instruments: the Maternal Antenatal Attachment Scale (MAAS) (26, 27), the Family Functioning Questionnaire (FFQ) (28), the Social Network Questionnaire (SNQ) (29). Data from the first screening performed for each participant, conducted at any point during the perinatal period (≥ 8 weeks of gestation), were analyzed.

All participating women provided informed consent to take part in the study. The add-on study was approved by the L’Aquila University Internal Review Board (n. 19/2022).

### Measures

Depressive symptoms during the 7 days preceding the assessment were evaluated using the Italian version of the Edinburgh Postnatal Depression Scale (EPDS) (25, 30), a 10-item self-report questionnaire assessing mood symptoms over the previous 14 days. Although two-factor structures (i.e., depression and anxiety) provide the best model fit in pregnancy samples (31), most of the reliable variance is accounted for by a general factor (32). Each item is scored from 0 to 3, yielding a total score between 0 and 30. A threshold of ≥ 12 is commonly used to identify clinically relevant depressive symptoms in perinatal populations (33). In our sample, the EPDS demonstrated strong psychometric properties, with McDonald’s omega total = 0.89.

Maternal–fetal attachment was assessed with the Italian version of the Maternal Antenatal Attachment Scale (MAAS) (26, 27). The MAAS is a 19-item self-report questionnaire that focuses specifically on the woman’s feelings, attitudes, and behaviors toward the fetus per se, rather than toward pregnancy or motherhood in general. Items are rated on a 5-point scale that reflects the frequency, intensity, or quality of feelings or behaviors experienced over the past 2 weeks. The MAAS comprises two dimensions: quality of attachment (MAAS-QA; 11 items), reflecting the valence and form of the emotional bond (e.g., pleasure in interaction, tenderness, distress at imagined loss, viewing the fetus as a “little person”), and intensity of preoccupation (MAAS-IP; 8 items), reflecting the amount of time and mental space devoted to the fetus (e.g., thinking, talking, dreaming about, or touching the baby through the abdomen). Scores are computed by summing the corresponding items (quality: 11–55; intensity of preoccupation: 8–40), with higher scores reflecting a more positive quality of attachment and greater intensity of preoccupation, respectively. In our sample, the MAAS demonstrated good psychometric properties, with McDonald’s omega total values of 0.87 and 0.75 for the quality of attachment and intensity of preoccupation subscales, respectively.

Family functioning was assessed using the Family Functioning Questionnaire (FFQ) (28). Developed to assess the pattern of family functioning, at the center of psychoeducational family interventions, the questionnaire consists of 24 items measuring the following 3 dimensions: (1) Problem solving (eight items), referred to the six steps of structured problem-solving: identify the problem or the objective, list possible alternative solutions, discuss the positive and negative aspects of each proposal, choose the best (or better, a satisfying, and realistic solution), plan the solution, check and review implementation and planning; (2) Communication skills (eight items), concerning the expression of positive and negative feelings, making of requests and active listening skills (probing questions, a summary of what has been understood), and (3) Personal Goals (eight items), defined as the ability of each family member to identify everyday personal goals (not linked to subject care). Responses range from 1 “never” to 4 “always.” Higher scores indicate healthier functioning. The items are evaluated on a 4-point Likert scale; higher scores indicate better family functioning (range 24–96). The scale was originally developed and standardized in the Italian population and has demonstrated good internal consistency (Cronbach’s alpha coefficients range from 0.75 to 0.84 across the three dimensions) and test–retest reliability (Pearson’s r coefficients range from 0.75 to 0.60) (28). Internal consistency for the FFQ in our sample was high (Cronbach’s a=0.88).

The Social Network Questionnaire (SNQ) (29) is a self-report questionnaire including 15 items, grouped based on factor analysis into four factors: (a) quality and frequency of social contacts; (b) practical social support; (c) emotional support; (d) quality of an intimate supportive relationship. Each item is rated on a four-point Likert scale, ranging from 1 = never to 4 = always. Internal consistency for the SNQ in our sample was high (Cronbach’s a=0.76).

Perceived emotional support from one’s life partner was evaluated with the 3-item Support Scale (34). The items are rated on a scale from 0 (no support) to 3 (high support). The partner support has been investigated in previous perinatal studies (5, 35–38), which consistently showed statistically significant associations with perinatal anxiety and mood conditions after adjustment for potential confounders.

### Statistical analysis

All analyses were performed in R version 4.5.1. We first computed descriptive statistics and Pearson correlations for family functioning (FFQ), depressive symptoms (EPDS), and the two attachment dimensions (MAAS): quality of attachment and intensity of preoccupation. To test the hypothesized mediation, we specified a multiple-outcome structural equation modeling (SEM) with the lavaan package (version 0.6–19) (39), in which FFQ predicted EPDS, which in turn predicted both MAAS subscales; direct paths from FFQ to each MAAS outcome were included to allow for partial mediation. A second model added perceived partner support and social network quality (QRS) as covariates predicting EPDS and both attachment outcomes. All models were estimated with maximum likelihood and full information maximum likelihood (FIML) for missing data, and standardized coefficients (β) are reported. Indirect effects via EPDS were evaluated using nonparametric bootstrapping with 5,000 resamples and percentile 95% confidence intervals. The strength of associations in correlations and standardized regressions was interpreted as follows: 0.00–0.19 indicates a very weak relationship, 0.20–0.39 a weak relationship, 0.40–0.59 a moderate relationship, 0.60–0.79 a strong relationship, and 0.80–1.00 a very strong relationship (40). Model fit was assessed using chi-square divided by degrees of freedom (χ²/df), Comparative Fit Index (CFI), Root Mean Square Error of Approximation (RMSEA), and Standardized Root Mean Square Residual (SRMR). Values of χ²/df ≤ 2, CFI ≥.90, RMSEA ≤.08, and SRMR ≤.08 are commonly treated as indicators of acceptable-to-good fit (41, 42), but these thresholds should be considered guidelines rather than strict cutoffs (31, 43).

## Results

### Participants

The study included 261 pregnant women, predominantly of Italian nationality, with a mean age of 33.8 years (SD = 4.9) and an average gestational age of 25.8 weeks (SD = 7).

Main socio-demographic and clinical characteristics of the sample are reported in **Table 1**.

**Table 1:** Socio-demographic and clinical characteristics of the sample.

Variables	Pregnant women (n=261)
Nationality, n (%)	
Italian	192 (73.6)
Educational level, n (%)	
Primary school diploma	1 (0.4)
Middle school diploma	10 (3.8)
High school graduation	118 (45.2)
University degree	132 (50.6)
Employment status, n (%)	
Housewife	28 (10.7)
Unemployed	33 (12.6)
Permanent employment	177 (67.8)
Precarious employment	17 (6.5)
Disability pension	1 (0.5)
Student	5 (1.9)
Marital Status, n (%)	
Married	233 (89.3)
Single	24 (9.2)
Divorced	4 (1.5)
Family status, n (%)	
Living with husband	253 (96.9)
Living with parents	6 (2.3)
Living independently	2 (0.8)
Socioeconomic status, n (%)	
Upper-middle status	102 (39.1)
Standard status	143 (54.8)
Low economic status	15 (5.7)
Several economic issues	1 (0.4)
Psychopharmacological treatment, n (%)	3 (1.1)
History of mental illness, n (%)	32 (12.3)
Past psychiatric diagnosis, n (%)	
Anxiety disorder	17 (53.3)
Major depressive disorder	11 (34.3)
Obsessive-Compulsive disorder	1 (3.1)
Post Traumatic Stress Disorder	1 (3.1)
Anorexia nervosa	1 (3.1)
Borderline Personality Disorder	1 (3.1)

Most participants were Italian, married, and living with their partner. Educational levels were generally high, with over half of the sample holding a university degree and more than 40% having completed high school. The majority of women were employed, primarily in permanent positions, while smaller proportions were unemployed or housewives.

Regarding socio-economic status, most participants reported a standard or upper-middle status. Only a small percentage reported current psychopharmacological treatment, while around 10% had a history of mental illness. Among those, anxiety disorders and major depressive disorder were the most frequently reported diagnoses.

Most of the women reported that their pregnancy was planned (**Table 2**). Nearly half of the participants had experienced at least one previous pregnancy. At the same time, a smaller proportion reported a history of abortion — only a limited number of women had conceived through medically assisted procreation. Additionally, a minority of the sample attended prenatal classes, suggesting moderate engagement in prenatal education.

**Table 2:** Clinical information related to the pregnancy.

Variables	Pregnant women (n=261)
Planned pregnancy, n (%)	
yes	183 (70.1)
no	78 (29.9)
Previous pregnancies, n (%)	
yes	121 (46.4)
no	140 (53.6)
Previous abortions, n (%)	
yes	73 (28)
no	188 (72)
Use of medically assisted procreation (MAP), n (%)	
yes	22 (8.4)
No	239 (91.6)
Prenatal classes, n (%)	
yes	83 (31.8)
no	178 (68.2)

**Figure 1:**
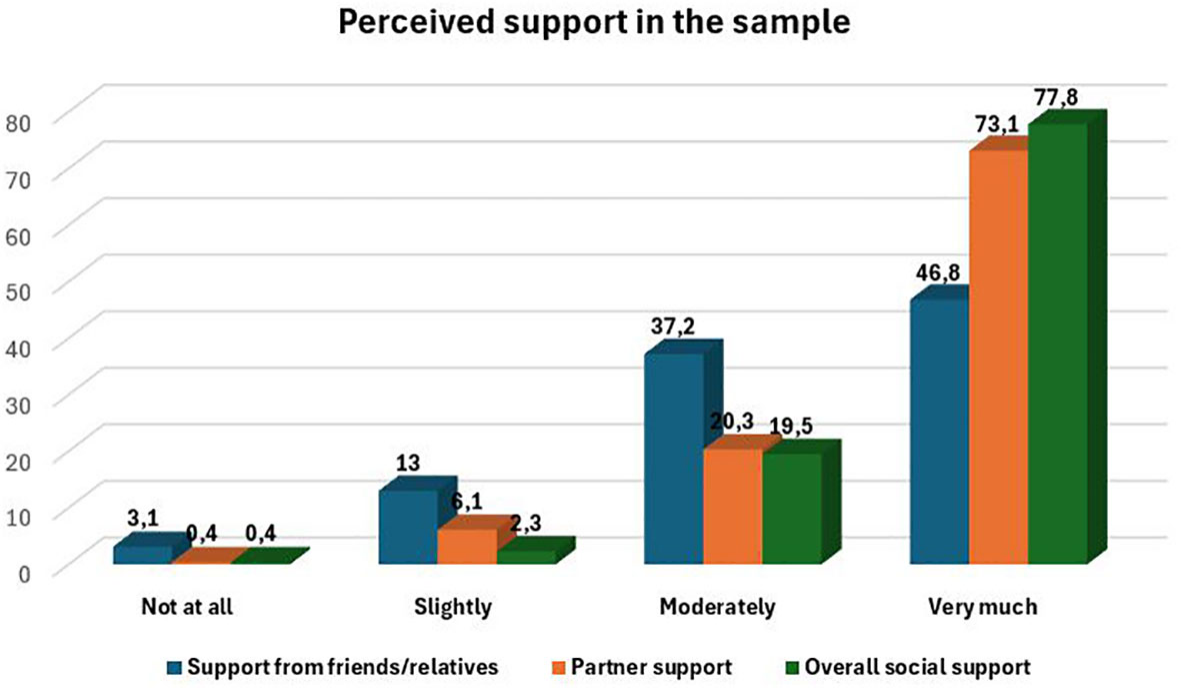
Perceived and reported social support among participants, as assessed through the 3-items Support Scale included in the socio-demographic questionnaire.

Most participants reported high levels of perceived social support (**Figure 1**). In particular, partner support and overall social support were predominantly rated as very high. In contrast, support from friends and relatives, although generally positive, showed slightly greater variability, with more responses in the moderate range. Only a small proportion of participants reported low levels of support across all sources.

### Descriptive statistics

Complete data were available for 261 pregnant women. The mean scores for the psychological measures were as follows: the Edinburgh Postnatal Depression Scale (EPDS) total score = 7.80 (SD = 3.68); the Maternal Antenatal Attachment Scale “Quality of Attachment” subscale = 50.66 (SD = 3.75) and “Intensity of Preoccupation” subscale = 30.69 (SD = 4.07); the Family Functioning Questionnaire (FFQ) total score = 70.91 (SD = 8.30); and the Social Network Questionnaire (QRS) total score = 0.80 (SD = 0.11).

Mean depressive symptoms were in the mild range (M = 7.8, SD = 3.7). Using the standard EPDS cutoff (≥ 12), 15.3% of women (n = 40) showed clinically relevant depressive symptoms.

Family functioning correlated weakly and negatively with depressive symptom severity (r = −.40, p <.001) and weakly and positively with attachment on both MAAS subscales (rs = .31 and.24, ps <.001). Depressive symptoms correlated weakly and negatively with attachment quality (r = −.29, p <.001) but did not correlate with attachment preoccupation (r = .01, p = .86). The two attachment facets were moderately intercorrelated (r = .56, p <.001).

### Model: depressive symptoms as mediator

The primary mediation model examined whether depressive symptoms (EPDS) mediated the association between family functioning (FFQ) and the two MAAS facets. As summarized in **Table 3**, family functioning was a moderate negative predictor of depressive symptoms (β = −.40, p <.001). In turn, depressive symptom severity showed a weak negative association with attachment quality (B = −.20, β = −.20, p <.05) and a very weak positive association with attachment preoccupation (B = .14, β = .13, p ≈.05) (see **Figure 1**).

**Table 3:** Indirect-path model (EPDS mediator) with and without covariates.

Path	Without covariates			With covariates		
	B	β	*p*-value	B	β	*p*-value
FFQ→ EPDS	−0.18	−.40	< .001	−0.15	−.34	< .001
EPDS → MAAS-QA	−0.20	−.20	.002	−0.20	−.19	.004
EPDS → MAAS-IP	0.14	.13	.487	0.15	.13	.440
FFQ → MAAS-QA (direct)	0.11	.23	.004	0.08	.18	.120
FFQ → MAAS-IP (direct)	0.14	.29	< .001	0.12	.24	.011
Indirect (FFQ → EPDS → QA)	0.04	.08	.002	0.03	.07	.011
Indirect (FFQ → EPDS → IP)	−0.03	−.05	.496	−0.02	−.05	.453
Total Indirect	0.01	.03	.146	0.01	.02	.219

Covariates, partner support and social network quality (QRS-100).

The direct effect of family functioning on both attachment facets remained weak but significant (β = .23 for quality, β = 0.29 for preoccupation), indicating partial mediation.

The indirect effect of family functioning → depressive symptoms → attachment quality was small and significant (βind = +.08). The total effect on attachment quality was.31, which means that ~26% of the total standardized effect was mediated. On the contrary, the indirect effect of family functioning → depressive symptoms → attachment preoccupation was negative and not significant.

Family functioning accounted for 16% of the variance in depressive symptoms, 18% of the variance in attachment quality, and 10% of the variance in preoccupation. Model fit indices were χ²/df = 2.03, CFI = .84, RMSEA = .06, and SRMR = .07.

### Model + covariates

**Figure 2:**
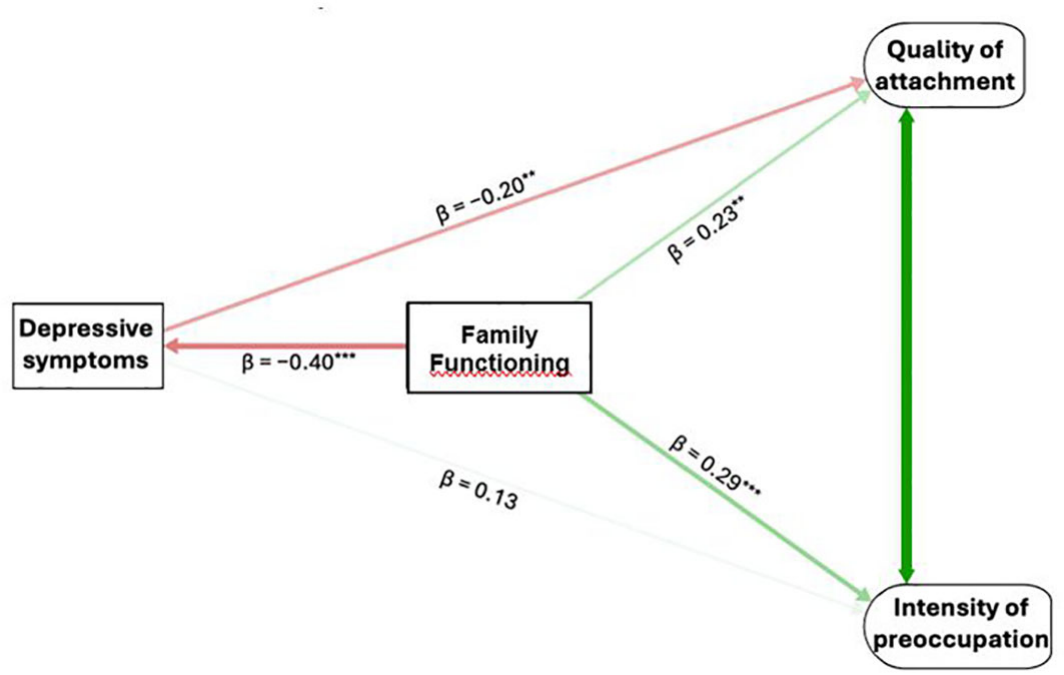
Structural model linking family functioning (FFQ), depressive symptoms (EPDS), and maternal antenatal attachment (MAAS). Standardized path coefficients (B) are shown from the EPDS-only multiple-outcome mediation model (no covariates). Higher family well-being is associated with fewer depressive symptoms (EPDS), which, in turks predict lower quality of attachment (MAAS-QA) and higher intensity of preoccupation (MAAS-IP), over and above the direct positive effects of family well-being on both attachment facets. Curved arrow indicates residual correlation between MAAS-QA and MAAS-IP.

When partner support and social network quality (QRS-100) were added as covariates, the overall pattern was unchanged. When partner support and social network quality (QRS-100) were added as covariates, the overall pattern was unchanged (**Figure 2**). Family functioning remained the strongest predictor of depressive symptoms (β = −.34, p <.001). The covariates did not show unique associations with depressive symptoms or the attachment outcomes (|β| ≤.10, ps >.10). Depressive symptoms predicted lower attachment quality (β = −.19, p = .004) but showed a non-significant positive relation with attachment preoccupation (β = .13, p = .44).

Direct effects from family functioning to the attachment facets were attenuated relative to the base model (Quality: β = .18, p = .12; Preoccupation: β = .24, p = .011).

Indirect effects via depressive symptoms were small: β = .07 (p = .011) for family functioning → depressive symptoms → attachment quality; β = −.05 (p = .453) for family functioning → depressive symptoms → attachment preoccupation. The total indirect effect was β = .02 (p = .219). This pattern indicates a significant indirect effect with a non-significant residual direct path for Quality (i.e., indirect-only mediation), and no mediation for Preoccupation.

Model fit with covariates was acceptable on absolute indices and modest on incremental indices (χ²/df = 1.85, CFI = .86, RMSEA = .06, and SRMR = .06).

## Discussion

The present study examined whether depressive symptoms mediate the association between family functioning and maternal–fetal attachment during pregnancy. The findings partially supported this hypothesis, indicating that mediation occurred for attachment quality but not for preoccupation intensity. Higher family functioning was consistently associated with lower levels of depressive symptoms, which in turn predicted only higher attachment quality. This pattern is consistent with prior research showing that more positive family functioning was associated with fewer depressive symptoms (20).

The indirect effect of family functioning on attachment quality through depressive symptoms was statistically significant, although very small in magnitude, indicating that better family functioning is linked to stronger emotional bonding primarily through reduced maternal psychological distress. Importantly, the modest size of this indirect effect is consistent with the multifactorial nature of prenatal attachment, family functioning, and maternal mental health. Maternal–fetal bonding is shaped by a complex interplay of biological, psychological, and contextual factors; therefore, indirect pathways are expected to account for only a limited proportion of the overall variance. In line with the family resilience framework, family functioning represents one of several interrelated protective factors operating within a broader system of influences (44). Accordingly, even small indirect effects may be clinically meaningful, as they reflect a specific mechanism within a network of cumulative and interacting risk and protective processes shaping maternal adaptation during pregnancy.

In contrast, no indirect effect was found for the preoccupation intensity dimension, as depressive symptoms were not significantly associated with it.

Instead, family functioning had a direct positive impact on the intensity of the preoccupation dimension, suggesting that women who perceive their family environment as more supportive and cohesive are more likely to think about the fetus and feel more cognitively involved, independently of depressive symptoms.

After controlling for perceived partner support and the quality of the social network, the overall mediation model remained stable: family functioning continued to predict lower depressive symptoms, which in turn predicted lower attachment quality. However, the direct path from family functioning to attachment quality became non-significant, indicating a purely indirect mediation for this dimension. Meanwhile, the relationship between family functioning and the intensity of preoccupation remained direct and significant, with no evidence of mediation.

These findings suggest that the emotional quality of prenatal attachment is more sensitive to maternal mood, whereas the cognitive–preoccupation component reflects broader relational factors within the family environment. This distinction aligns with conceptual models that distinguish between affective warmth and cognitive investment in the maternal–fetal bond (45). The fact that depressive symptoms are unrelated to the intensity of preoccupation, but inversely associated with attachment quality, supports the idea that distress primarily undermines emotional closeness and, if at all, only secondarily affects cognitive involvement.

The lack of unique effects for partner support and social network quality indicates that family functioning captures broader aspects of the family climate—such as cohesion, communication, and shared values—that go beyond dyadic or social support alone. This highlights the distinct and fundamental role the family environment plays in shaping maternal emotional functioning during pregnancy.

## Clinical implications

Our findings suggest that promoting family functioning may indirectly enhance the quality of maternal–fetal attachment by reducing depressive symptoms. Prenatal programs aimed at improving family communication and emotional support from partners could yield psychological benefits that extend to prenatal bonding. At the same time, psychosocial interventions for pregnant women should take into account that cognitive engagement with the fetus depends more on the overall family connection than on maternal mood. An assessment of family dynamics should accompany routine screening for depressive symptoms (e.g., using the EPDS).

## Strengths and limitations

This study has several strengths, including the use of a dual-outcome SEM approach that allowed simultaneous examination of both affective and cognitive components of maternal–fetal attachment. In addition, the use of bootstrapped indirect effects and full-information maximum likelihood estimation enhances the robustness and reliability of the findings.

However, several limitations should be acknowledged. First, the cross-sectional design limits the ability to infer directionality or causality from the observed associations. Although the mediation model is theoretically grounded, longitudinal studies are needed to confirm temporal relationships among family functioning, depressive symptoms, and maternal–fetal attachment.

Second, an *a priori* power analysis was not conducted for the SEM model. Although the sample size may be considered adequate, the absence of a formal power estimation limits the ability to determine whether the study was sufficiently powered to detect small effects, particularly for indirect pathways.

Third, partner support was assessed using a single item from the 3-item Support Scale, which may limit both reliability and the depth of construct assessment. Future studies would benefit from using multi-item, validated instruments to better capture the complexity of perceived partner support.

Fourth, the sample may have limited representativeness and may be subject to selection bias. Participants appeared relatively socioeconomically stable, with most reporting stable economic conditions, cohabitation with a partner, and high levels of perceived partner support. This restricted variability may have attenuated the observed associations and limits the generalizability of the findings to more socioeconomically vulnerable or high-risk populations.

Finally, the modest proportion of explained variance in the outcome variables suggests that additional factors—such as biological stress markers, partner mental health, or broader contextual influences—may also play a significant role and should be considered in future research.

## Conclusions

Family functioning contributes to maternal–fetal attachment through distinct pathways, highlighting the importance of differentiating between the emotional and cognitive components of the bond. Better family functioning is associated with fewer maternal depressive symptoms, which in turn are linked to stronger emotional attachment but not to increased preoccupation with the fetus. The emotional quality of prenatal attachment thus appears more sensitive to maternal mood, whereas the intensity of preoccupation reflects broader relational dynamics within the family context. Overall, our findings underscore the central role of the family climate in shaping maternal emotional functioning during pregnancy. Interventions that foster a supportive and cohesive family environment and reduce maternal distress may promote healthier prenatal bonding and potentially enhance early parent–child relationships.

## Data Availability

The raw data supporting the conclusions of this article will be made available by the authors, without undue reservation.
